# Occurrence of the birds of the Middle Volga Region (South-East of the European part of Russia)

**DOI:** 10.3897/BDJ.9.e72075

**Published:** 2021-11-03

**Authors:** Anastasia Klenina, Alexander Ruchin, Evgenii Bykov

**Affiliations:** 1 Samara Federal Research Center of Russian Academy of Sciences, Institute of Ecology of the Volga River basin of Russian Academy of Sciences, Tollyatti, Russia Samara Federal Research Center of Russian Academy of Sciences, Institute of Ecology of the Volga River basin of Russian Academy of Sciences Tollyatti Russia; 2 Mordovia State Nature Reserve and National Park «Smolny», Saransk, Russia Mordovia State Nature Reserve and National Park «Smolny» Saransk Russia

**Keywords:** dataset, bird occurrences, avifauna, Aves, data paper

## Abstract

**Background:**

Birds are the most numerous and widespread group of higher vertebrates. Due to the peculiarities of their biology, birds play an important role in nature and in human life.

Ornithological studies described in this publication were conducted in seven regions of the Middle Volga Region (Chuvashia, Mordovia, Tatarstan, Samara, Nizhny Novgorod, Ulyanovsk and Penza Regions) from 1978 to 2021. Visual and acoustic methods were used to study the species composition during field studies. In total, 5065 birdoccurrences belonging to 157 species, 48 families and 19 orders were registered. All occurrences have a geographical reference. The large volume of data collected, the wide geographical coverage and the long-term nature of the observations determined the value of their inclusion in the GBIF and the need for publication in the Biodiversity Data Journal.

**New information:**

We are publishing our original data on the coordinates of bird occurrences in the Middle Volga Region for the first time. Most of the original information about bird occurrences was contained in field diaries and was not available to a wide range of researchers. All 5065 occurrences are new to GBIF.

## Introduction

Birds play an important role in natural complexes and in human life. They minimise outbreaks of harmful insects, regulate the number of rodents and dispersal of seeds of several plants (Raman 2001, Rey Benayas et al. 2017, Carvalho et al. 2020, Simonov and Matantseva 2020). They can serve as bioindicators for the state of the environment, since they respond to anthropogenic impact by changing the species composition of nesting avifauna and the number of various ecological groups (Bykov 2013, Lebedinskii et al. 2019, Kuznetsova 2021). In addition, birds make a significant contribution to the spread of parasites and transmissible diseases and can partially destroy grain crops (Girikaran et al. 2019, Stanković et al. 2019, Kucherenko et al. 2020). It shows the need for a comprehensive study of this animal group.

The avifauna of the Middle Volga Region is quite well studied, but it is still relevant to conduct new research since there are migratory species and species expanding their range (Lebedeva 2017). Thus, over the past 20 years, the appearance of such new species as *Phoenicurusochruros* (S. G. Gmelin, 1774) and *S.decaocto* (Frivaldszky, 1838) has been noted for the Samara Region, including cases of their nesting (Lebedeva 2017, Klenina et al. 2021). *Streptopeliadecaocto* (Frivaldszky, 1838) and *S.alba* deepened their connection with the territory as, previously, they they were only registered (Lebedeva 2017, Klenina et al. 2021).

### Protection status

Several registered species are listed in the Red Data Lists of different levels. Thus, the Red Book of the Russian Federation (Ministry of Nature Resources and Ecology of the Russian Federation 2020) includes species caught in the Middle Volga Region: *Otistarda* Linnaeus, 1758, *Pandionhaliaetus* (Linnaeus, 1758), *Buteorufinus* (Cretzschmar, 1829), *Aquilanipalensis* Hodgson, 1833, *Aquilachrysaetos* (Linnaeus, 1758), *Aquilaheliaca* Savigny, 1809, *Haliaeetusalbicilla* (Linnaeus, 1758), *Falcocherrug* J.E. Gray, 1834, *Falcoperegrinus* Tunstall, 1771, *Anthropoidesvirgo* (Linnaeus, 1758), *Tetraxtetrax* (Linnaeus, 1758), *Haematopusostralegus* Linnaeus, 1758 (mainland subspecies); *Bubobubo* (Linnaeus, 1758). In the IUCN Red List (International Union for Conservation of Nature and Natural Resources 2021), *A.nipalensis* and *Falcocherrug* are ranked as Endangered (EN); *Streptopeliaturtur* (Linnaeus, 1758), *Aythyaferina* (Linnaeus, 1758), *A.heliaca*, *O.tarda* ranked as Vulnerable (VU); and *Vanellusvanellus* (Linnaeus, 1758), *Turdusiliacus* Linnaeus, 1758, *H.ostralegus* and *T.tetrax* ranked as Near Threatened (NT). Other bird species are ranked as Least Concern (LC) (International Union for Conservation of Nature and Natural Resources 2021). Twenty-seven species from the Red Book were found in the Samara Region (Simak et al. 2019). 5 species also being found in the Republic of Mordovia (Astradamov 2005).

## Project description

### Title

Occurrence of the birds of the Middle Volga Region (South-East of the European part of Russia)

### Study area description

Middle Volga Region

## Sampling methods

### Sampling description

The dataset is based on original data obtained during field observations of birds in the period from 1978 to 2021 in the Middle Volga Region. The study of avifauna was carried out within the following administrative regions of the Russian Federation: the Republics of Chuvashia, Mordovia and Tatarstan; in the Samara, Nizhny Novgorod, Ulyanovsk and Penza Regions. The study of the species composition of birds was carried out in the course of field research, including in the framework of quantitative accounting for the number of birds. The number of birds according to the mating song of the male was recorded during the nesting period according to the standard method (Priednieks et al. 1986). Sometimes visual identification of the species belonging to a singing bird was carried out using a bird identifier (Flint et al. 1968). The species were identified on the basis of their vocalisation or by using a visual method with the help of the keys released at the time of observation (Promptov 1929, Ryabitsev 2008, Kalyakin and Voltzit 2020). If there were feather residues, an atlas for feather identification was used (Korepova 2016). In cases where it was difficult to determine a bird to a species in the field due to minor differences in plumage or vocalisation, we resorted to additional actions (for example, photographing and looking for nests). If the definition were impossible, it was not entered into the database. Some of the data were collected during special bird observations, during route surveys of birds of recreational forests (Bykov 2000, Bykov 2016), as well as during the study of the effectiveness of bird protection devices on power lines (Klenina and Bakiev 2014).

### Step description

Field research included the determination of the species composition of birds of the region by audio-visual methods, with a record of the date of the meeting of the species and the coordinates of its habitat. To make the geographical reference, we fixed the coordinates of the location of the bird's occurrence using a GPS navigator or using Google maps. In all cases, the WGS-84 coordinate system was used with the accuracy of determining the coordinates up to the fourth digit. If the bird could not be detected visually, then its identification was made by listening to its voсalisation, the place of occurrence was then the place where the bird was heard. Since the bird class is one of the most geographically mobile from all the taxonomic groups of animals, this assumption seems insignificant. Subsequent processing of materials included entering data into an Excel spreadsheet and preparing a dataset in accordance with the Darwin core format, uploading the dataset to GBIF.

The field names of the dataset were chosen according to Darwin Core (Wieczorek et al. 2012).

## Geographic coverage

### Description

The dataset contains information about the occurrence of birds in seven administrative Regions of the Russian Federation: the Chuvash Republic, the Republic of Tatarstan, the Republic of Mordovia, Penza, Samara, Ulyanovsk and Nizhny Novgorod Regions. These Regions are located in the European part of Russia, which is designated by the Russian Bird Conservation Union as a key ornithological territory. It is used by birds as nesting sites, moulting, wintering and stopover sites (Sviridova and Zubakin 2000). Ornithological studies were conducted within the territory of such protected areas as the Mordovia State Nature Reserve (Republic of Mordovia), the national parks “Samarskaya Luka” (Samara Region) and “Smolny” (Republic of Mordovia).

**Figure 1.**
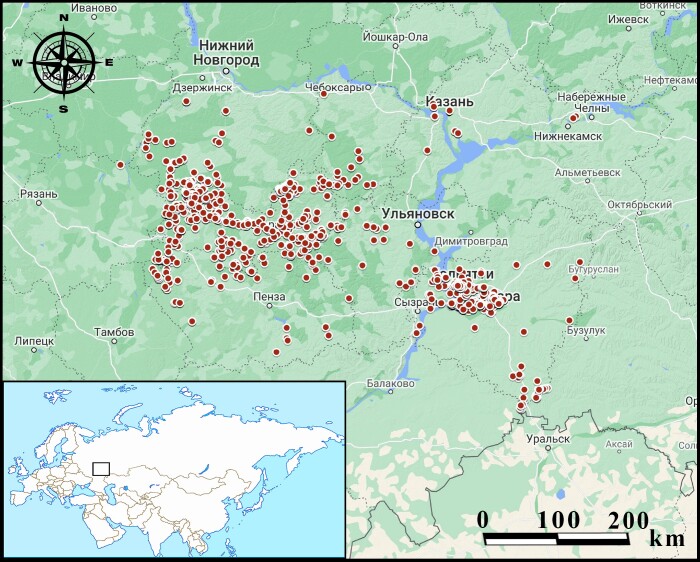
The occurrences of birds (red circles) within the studied area of the Middle Volga Region.

The studied territory is in the south-east of the European territory of Russia in the middle course of the Volga River (Fig. 1).

### Coordinates

51.8 N and 56.0 N Latitude; 41.3 E and 52.0 E Longitude.

## Taxonomic coverage

### Description

**Figure 2.**
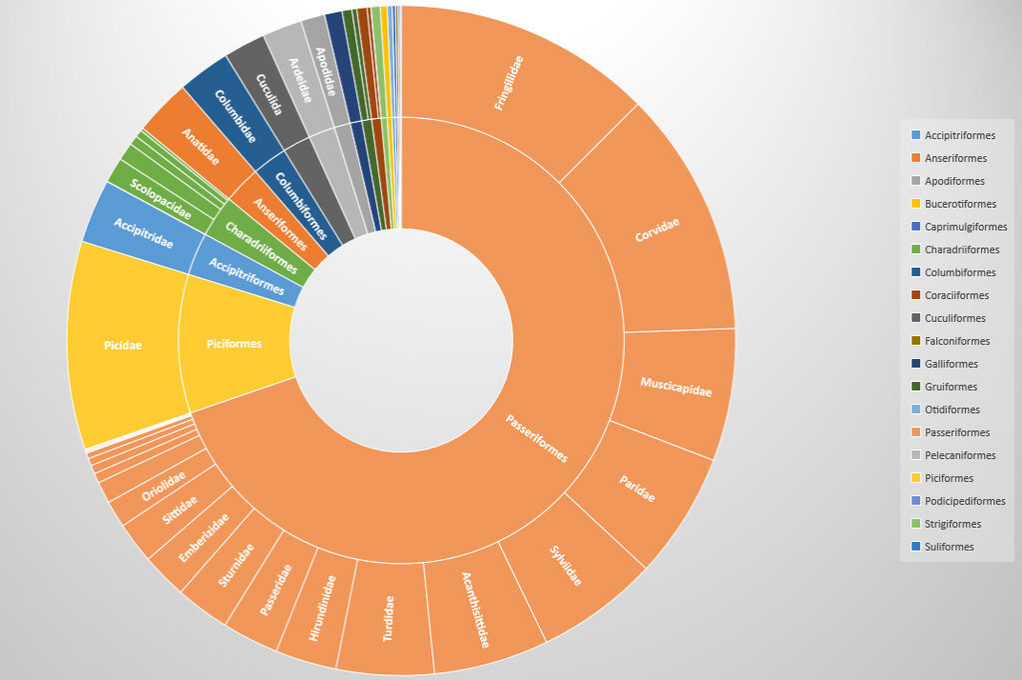
Taxonomic coverage of registered species by orders and families.

The dataset includes only occurrences of birds identified to the species level. In total, 157 species belonging to 48 families from 19 orders have been registered (Fig. 2). It is about a third of the species composition of the avifauna of the European territory of Russia (Kalyakin and Voltzit 2020). The noted species belong to 48 families from 19 orders (see Table of Taxa included below). According to our data, the most common in the Middle Volga Region are *Fringillacoelebs* (451 occurrences of 768 individuals), *Dendrocoposmajor* (381 occurrences of 578 individuals) and *Parusmajor* (260 occurrences of 459 individuals) (Table 1).

### Taxa included

**Table:** 

Rank	Scientific Name	
order	Accipitriformes	
order	Anseriformes	
order	Apodiformes	
order	Bucerotiformes	
order	Caprimulgiformes	
order	Charadriiformes	
order	Columbiformes	
order	Coraciiformes	
order	Cuculiformes	
order	Falconiformes	
order	Galliformes	
order	Gruiformes	
order	Otidiformes	
order	Passeriformes	
order	Pelecaniformes	
order	Piciformes	
order	Podicipediformes	
order	Strigiformes	
order	Suliformes	

## Usage licence

### Usage licence

Other

### IP rights notes

Creative Commons Attribution (CC-BY) 4.0 License

## Data resources

### Data package title

Occurrence of the birds of the Middle Volga Region (south-East of the European part of Russia)

### Resource link

https://www.gbif.org/dataset/b93ec686-b0c6-40d7-a7bb-e0f505bbd0e9

### Alternative identifiers

https://doi.org/10.15468/y46dzf

### Number of data sets

1

### Data set 1.

#### Data set name

Occurrence of the birds of the Middle Volga Region (south-East of the European part of Russia)

#### Data format

Darwin Core Archive format

#### Number of columns

19

#### Character set

UTF-8

#### Download URL

https://www.gbif.org/occurrence/download?dataset_key=b93ec686-b0c6-40d7-a7bb-e0f505bbd0e9

#### Description

Our occurrence record contains the occurrence ID, basis of record, species name, taxonomic characteristic, geographic coordinates, event date and authors of the record and species identification. All occurrence records are georeferenced. The dataset is based on research by the staff of the Institute of Ecology of the Volga River Basin of the Russian Academy of Sciences and of the Mordovia State Nature Reserve and National Park «Smolny».

**Data set 1.** 

Column label	Column description
occurrenceID	An identifier for the bird occurrence. https://dwc.tdwg.org/terms/#dwc:occurrenceID
basisOfRecord	The specific nature of the data record. https://dwc.tdwg.org/terms/#dwc:basisOfRecord
scientificName	The full scientific name of bird, with authorship and date information. https://dwc.tdwg.org/terms/#dwc:scientificName
kingdom	The full scientific name of the kingdom in which the taxon is classified. https://dwc.tdwg.org/terms/#dwc:kingdom
phylum	The full scientific name of the phylum or division in which the taxon is classified. https://dwc.tdwg.org/terms/#dwc:phylum
class	The full scientific name of the class in which the taxon is classified. https://dwc.tdwg.org/terms/#dwc:class
order	The full scientific name of the order in which the taxon is classified. https://dwc.tdwg.org/terms/#dwc:order
family	The full scientific name of the family in which the taxon is classified. https://dwc.tdwg.org/terms/#dwc:family
geodeticDatum	The ellipsoid, geodetic datum or spatial reference system (SRS) upon which the geographic coordinates given in decimalLatitude and decimalLongitude are based. https://dwc.tdwg.org/terms/#dwc:geodeticDatum
coordinateUncertaintyInMetres	The horizontal distance (in metres) from the given decimalLatitude and decimalLongitude describing the smallest circle containing the whole of the Location. https://dwc.tdwg.org/terms/#dwc:coordinateUncertaintyInMeters
coordinatePrecision	A decimal representation of the precision of the coordinates given in the decimalLatitude and decimalLongitude. https://dwc.tdwg.org/terms/#dwc:coordinatePrecision
decimalLatitude	The geographic latitude of the geographic centre of a Location. https://dwc.tdwg.org/terms/#dwc:decimalLatitude
decimalLongitude	The geographic longitude of the geographic centre of a Location. https://dwc.tdwg.org/terms/#dwc:decimalLongitude
country	The name of the country in which the Location occurs. https://dwc.tdwg.org/terms/#dwc:country
countryCode	The standard code for the country in which the Location occurs. https://dwc.tdwg.org/terms/#dwc:countryCode
individualCount	The number of individuals represented present at the time of the Occurrence. https://dwc.tdwg.org/terms/#dwc:individualCount
eventDate	The date when the event was recorded. https://dwc.tdwg.org/terms/#dwc:eventDate
recordedBy	A person who responsible for recording the original Occurrence. https://dwc.tdwg.org/terms/#dwc:recordedBy
identifiedBy	A person who assigned the Taxon to the subject. https://dwc.tdwg.org/terms/#dwciri:identifiedBy

## Figures and Tables

**Table 1.** The top 20 species of the dataset ordered by the number of observations.

Rank	Species	Number of observations
1	*Fringillacoelebs* Linnaeus, 1758	451
2	*Dendrocoposmajor* (Linnaeus, 1758)	381
3	*Parusmajor* Linnaeus, 1758	260
4	*Corvuscornix* Linnaeus, 1758	193
5	*Motacillaalba* Linnaeus, 1758	180
6	*Turduspilaris* Linnaeus, 1758	167
7	*Picapica* (Linnaeus, 1758)	146
8	*Sturnusvulgaris* Linnaeus, 1758	133
9	*Milvusmigrans* (Boddaert, 1783)	107
10	*Cuculuscanorus* Linnaeus, 1758	103
11	*Sittaeuropaea* Linnaeus, 1758	101
12	*Emberizacitrinella* Linnaeus, 1758	94
13	*Ardeacinerea* Linnaeus, 1758	93
14	*Anthustrivialis* (Linnaeus, 1758)	91
15	*Corvusfrugilegus* Linnaeus, 1758	91
16	*Passermontanus* (Linnaeus, 1758)	85
17	*Chlorischloris* (Linnaeus, 1758)	76
18	*Coloeusmonedula* (Linnaeus, 1758)	75
19	*Phylloscopuscollybita* (Vieillot, 1817)	74
20	*Anasplatyrhynchos* Linnaeus, 1758	73
